# Whole Genome Analysis of a Community-Associated Methicillin-Resistant *Staphylococcus aureus* ST59 Isolate from a Case of Human Sepsis and Severe Pneumonia in China

**DOI:** 10.1371/journal.pone.0089235

**Published:** 2014-02-20

**Authors:** Tingting Qu, Ye Feng, Yan Jiang, Peiqiong Zhu, Zeqing Wei, Yan Chen, Michael Otto, Yunsong Yu

**Affiliations:** 1 State Key Laboratory for Diagnosis and Treatment of Infectious Disease, First Affiliated Hospital, College of Medicine, Zhejiang University, Hangzhou, Zhejiang, China; 2 Department of Infectious Diseases, Sir Run Run Shaw Hospital, College of Medicine, Zhejiang University, Hangzhou, Zhejiang, China; 3 Department of Clinical Medicine, Zhejiang Medical College, Hangzhou, Zhejiang, China; 4 Pathogen Molecular Genetics Section, Laboratory of Human Bacterial Pathogenesis, National Institute of Allergy and Infectious Diseases, National Institutes of Health, Bethesda, Maryland, United States of America; Rockefeller University, United States of America

## Abstract

We report a case of necrotizing pneumonia in a young patient caused by community acquired-methicillin resistant *Staphylococcus aureus* (CA-MRSA) in a teaching hospital in the People’s Republic of China. The patient had a typical clinical presentation and was successfully treated with antibiotics and intravenous immunoglobulin. A CA-MRSA strain, named SA268, was isolated from the blood of the patient. The isolate was susceptible to most antimicrobial agents, except cephalosporins, penicillins, and β-lactamase inhibitor combinations. Multi-locus sequence typing (MLST) assigned SA268 to ST59, a clone widely spread in eastern Asia. The strain was positive for Panton Valentine Leukocidin (PVL)-encoding genes and SCC*mec* type V. We sequenced the complete genome of the SA268 isolate. The genome of SA268 was almost identical to that of the Taiwanese ST59 CA-MRSA strains M013 and SA957. However, we observed several differences in gene composition, which included differences in the SCC*mec* element and several lipoprotein genes that were present in the Taiwanese strains but absent from SA268.

## Introduction

While infections with methicillin-resistant *Staphylococcus aureus* (MRSA) have traditionally been limited to hospitals, community-associated cases of MRSA (CA-MRSA) were reported starting in the late 1990s [1], [2]. CA-MRSA strains have the capacity to infect healthy individuals outside of hospital settings; and CA-MRSA infections are frequently observed in children, young adults, and the elderly. The clinical manifestations of CA-MRSA infections mostly include skin and soft tissue infections (SSTIs) and occasionally life-threatening invasive infections, such as sepsis, necrotizing pneumonia and osteomyelitis [2], [3].

The first well documented CA-MRSA cases appeared in the upper midwestern United States between 1997 and 1999 in children [1]. These infections, which were fatal cases of sepsis and severe pneumonia, were mostly caused by strain MW2 (pulsed-field type USA400). Later, strains of pulsed-field type USA300 gradually replaced USA400 strains in the U.S. [4], although USA400 CA-MRSA infections can still be observed in Alaska [5]. While the U.S. has experienced the most pronounced CA-MRSA epidemic, CA-MRSA has also been a global problem. Currently there are five dominant CA-MRSA lineages: ST1-IV, ST8-IV, ST30-IV, ST59-IV/V, and ST80-IV, each being prevalent in distinct geographical locations [6]. CA-MRSA lineages have generally been characterized by carriage of small-sized staphylococcal cassette chromosome *mec* elements (SCC*mec*) of types IV and V, and in many cases, presence of the genes encoding Panton-Valentine leukocidin (PVL) [7].

The epidemiological success of CA-MRSA strains is believed to stem from the combination of antibiotic resistance at a low fitness cost with extraordinary virulence, allowing these strains to infect otherwise healthy individuals and spread sustainably in the community [7], [8], [9]. However, research on CA-MRSA virulence has been performed largely with the USA300 lineage, whereas our knowledge on virulence of other CA-MRSA lineages is relatively limited.

ST59 is a major MRSA clone in eastern Asia [10]. CA-MRSA isolates of ST59 have been reported in Taiwan, which can be distinguished from the commensal ST59 Asian-Pacific MRSA clone by the presence of PVL-encoding genes, absence of the prophage that inserts into the β-toxin gene *hlb*, and an increased expression of core genome-encoded virulence factors such as α-toxin [11], [12]. ST59 CA-MRSA isolates thus have an increased virulence potential as compared to their commensal ST59 counterparts. Two genomes of Taiwanese ST59 CA-MRSA isolates, strains SA957 and M013, have been published [11], [12]. In contrast, knowledge on CA-MRSA in the People’s Republic of China (mainland China) is limited and has been mostly obtained for infections in children. According to these studies, ST59 is the most frequent clone among Chinese CA-MRSA isolates [10], [13], [14]. The complete genome of a CA-MRSA isolate from the People’s Republic of China has not been reported yet.

In this study, we describe a case of sepsis and severe pneumonia caused by a ST59 CA-MRSA strain in a teaching hospital in the Zhejiang province of China. We sequenced the whole genome of this strain and compared it with other major CA-MRSA lineages focusing on virulence factors and genomic/pathogenicity islands.

## Materials and Methods

### Strain Isolation and Ethics Statement

The MRSA strain SA268 was isolated from both the blood and the sputum of a young patient with severe sepsis and acute respiratory failure in March of 2012. Written informed consent was obtained from the patient’s mother. The isolate was stored in the Department of Microbiology, the First Affiliated Hospital, College of Medicine, Zhejiang University. We obtained an exempt status from the Institutional Review Board of the First Affiliated Hospital, College of Medicine, Zhejiang University to use the strain for all experiments in this study.

### Antibiotic Resistance

Susceptibility testing for SA268 was performed using the Etest strip according to the manufacturer’s instructions. Results were interpreted according to the recommendations and definitions from the Clinical and Laboratory Standards Institute (CLSI). Antimicrobial agents tested included penicillin G, ampicillin/sulbactam, cefoxitin, gentamicin, vancomycin, teicoplanin, levofloxacin, sulfamethoxazole-trimethoprim, linezolid, tigecycline, daptomycin, tetracycline, fosfomycin and rifampin. ATCC29213 (*S. aureus*) and ATCC29212 (*Enterococcus faecalis*) were used as quality controls.

### PFGE and MLST Analysis

Bacterial DNA was digested with *Sma*I and the digested DNA was applied to pulsed-field gel electrophoresis (PFGE) on 1.2%-agarose gels, as described previously [34]. Multi-locus type sequencing (MLST) was performed as described previously comparing sequences of the PCR products against the MLST website (http://saureus.mlst.net) [15].

### Genome Sequencing and Annotation

The genomic DNA of SA268 was extracted using a QIAamp DNA Mini Kit (Qiagen, CA, USA). The TruSeq® DNA Sample Preparation Kit was used for constructing the pair-end and mate-pair sequencing library and then the libraries were launched on Illumina® Hiseq2000 sequencer (Illumina, CA, USA). The average fragment sizes for the pair-end and mate-pair libraries were 300 bp and 3,000 bp, respectively. The read length was 100 bp. The velvet assembler v1.0 program was used to assemble the Illumina reads [16]. The Columbus module was used and the genome of strain M013 (accession number CP003166.1) was used as the reference sequence.

The derived contigs were compared with the genome of strain M013 by MUMmer software [17] to determine the relationships between contigs. Gaps between contigs were closed by PCR amplification and sequencing.

The genome sequences of the chromosome and the plasmid were submitted to the web service RAST for automatic annotation followed by manual evaluation [18].

### Genomic Comparison and Phylogenetic Analysis

Similarities of protein-coding sequences were determined using the blastn program of the NCBI Basic Local Alignment Search Tool (BLAST). For a coding sequence to be considered homologous, the DNA identity had to be greater than 80%, the e-value smaller than 1e-10, and the aligned length larger than 80% of the gene sequence.

For phylogenetic comparison within ST59, the genome sequences of strains SA268, M013, SA957, MW2, FPR3757 and 11819-97 were imported into the MAUVE program [19]. The derived backbone regions, namely regions that are conserved in all these strains, were extracted and were further aligned by the MAFFT program [20]. The aligned sequences were imported into the PhyML program [21]. A maximum likelihood tree was produced based on the JC69 substitution model, and the SH-like support value was calculated. The number of pair-wise single nucleotide substitutions between strains was calculated from the backbone regions with a script made in our institute.

### Nucleotide Sequence Accession Number

The genome of SA268 was deposited in the NCBI database under the accession number CP006630 (chromosome) and KF471116 (plasmid). The accession numbers for the genomes of strains FPR3757, MW2, 11819-97, M013, SA957 and pPM1 are CP000255.1, BA000033.2, CP003194.1, CP003166.1, CP003603.1, AB699881.1, respectively.

## Results and Discussion

### Case Report

**Figure 1:**
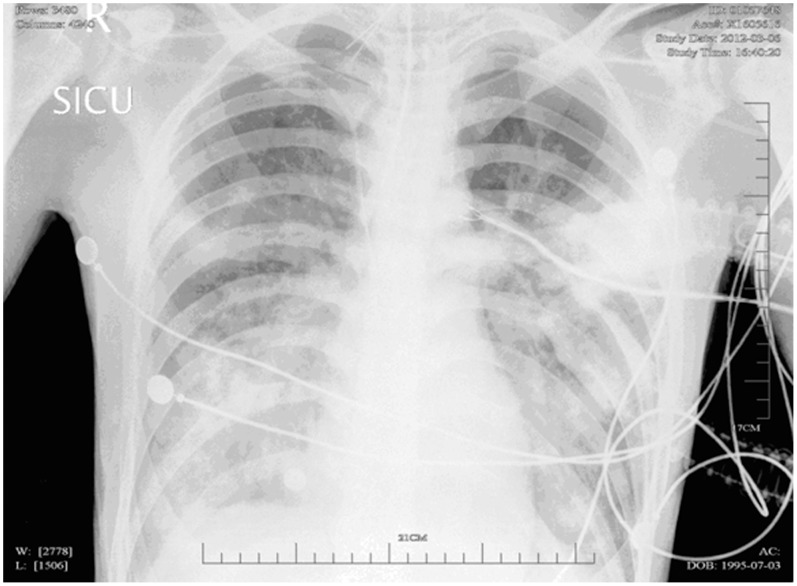
Chest radiograph on admission showing bilateral dense infiltrates.

**Figure 2:**
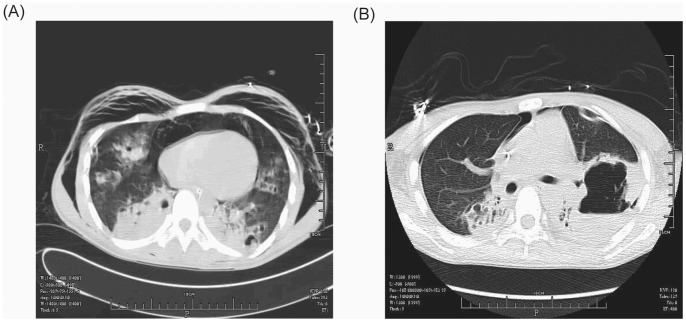
Lung CT scan on (A) day 7 of hospitalization showing abscess formations and (B) day 26 of hospitalization showing a large abscess cavity with a fluid level in the left lobe.

A 16-year-old, previously healthy, Chinese male presented with severe sepsis and acute respiratory failure. He had never travelled to Taiwan or other countries. In the week before hospitalization, he noted an influenza-like illness. On examination, chest auscultation revealed discrete inspiratory crackles over the lower lung fields. The chest radiograph showed bilateral dense alveolo-interstitial infiltrates predominantly in the middle and lower lobes (Figure 1). The leukocyte count was 1.2 G/l, with a neutrophil ratio of 72.5%. The C-reactive protein count was 163 mg/l. Other laboratory parameters were normal. Within the first 24 hours, hypoxemia worsened (PaO_2_/FiO_2_<100 mm Hg), profound septic shock developed, and the leukocyte count dropped to 0.82 G/l. Extracorporeal membrane oxygenation was used for six days. Sputum and blood culture both grew MRSA. The HIV test was negative. PCR amplification performed on a throat swab was positive for influenza B. The patient remained febrile, and a computed tomography (CT) scan on the 7^th^ day revealed extensive infiltrations with cavitations suggestive of multiple abscesses, pneumothorax and subcutaneous emphysema (Figure 2a). The patient was treated with teicoplanin (400 mg twice daily for 3 days, followed by 400 mg once daily for 6 days), and then was switched to linezolid (600 mg twice daily). High-dose intravenous immunoglobulin (2 g/kg) was added for four days. The CT scan on the 26^th^ day revealed a large abscess cavity with a fluid level (Figure 2b). The patient required prolonged mechanical ventilation and antibiotic therapy because of abscess development and several episodes of acute respiratory distress. Teicoplanin and linezolid treatment lasted 29 and 15 days, respectively. The patient was successfully weaned after 49 days of mechanical ventilation and was transferred to the medical ward after 76 days in the intensive care unit. He was discharged from the hospital after five months.

### Genetic Typing and Complete Genome Sequencing

The MRSA isolates obtained from the blood and sputum of the patient exhibited the same PFGE pattern (Figure S1) and the same MLST type (ST59). These typing results demonstrated that the two isolates belonged to be the same clone. We selected the blood isolate (named SA268) for complete genome sequencing. Sequencing by Illumina Hiseq produced the draft genome, with the average coverage over 500-fold. A total of 17 contigs had their size larger than 1 kb. The maximal contig was 959707 bp long, the N50 size was 854282 bp, and the N90 size was 181768 bp. Gaps between contigs were then closed by PCR amplification and sequencing. Finally, we obtained the complete sequence of a chromosome and a plasmid, the genetic information of which was summarized in Table 1.

**Table 1:** General features of the CA-MRSA SA268 genome and the pSA268 plasmid.

Element and characteristic	Value
Chromosome	
Size (bp)	2,833,899
Coding regions (%)	84.2
G+C content (%)	32.9
Protein-coding sequences	2656
tRNA genes	60
rRNA operons	5
Plasmid pSA268	
Size (bp)	20,269
Coding regions (%)	67.1
G+C content (%)	28.6
No. of protein-coding sequences	22

### Susceptibility Profiles

As shown in Table 2, we found that SA268 was resistant to penicillins, β-lactamase inhibitor combinations, and susceptible to aminoglycosides, quinolones, teicoplanin, vancomycin, tetracycline, fosfomycin, rifampicin, trimethoprim/sulfamethoxazole, linezolid, tigecycline and daptomycin.

**Table 2:** Susceptibility of SA268 to antimicrobial agents.

Antimicrobial agent	MIC (µg/ml)
Cefoxitin	32
Linezolid	0.5
Sulfamethoxazole-trimethoprim	0.047
Gentamicin	0.38
Vancomycin	0.75
Ampicillin/Sulbactam	4
PenicillinG	16
Teicoplanin	1
Levofloxacin	0.19
Tetracycline	0.38
Tigecycline	0.094
Fosfomycin	<0.064
Rifampicin	0.004
Daptomycin	0.125

### Genomic and Pathogenicity Islands

A comparison of genomic and pathogenicity islands of strain SA268 to the main global CA-MRSA lineages is shown in Table 3. SA268 harbors νSaα, νSaβ and ΦSa2. The genomic islands νSaα and νSaβ occur in almost all *S. aureus* strains. Nevertheless, νSaβ of ST59 is different from that found in other *S. aureus* lineages. νSaβ usually contains a type I restriction/modification system and may also carry genes encoding virulence factors, such as the bicomponent leukotoxin LukDE, serine proteases, enterotoxins, and an epidermin-like bacteriocin. However, these virulence genes are absent from ST59 strains. The PVL-encoding prophage ΦSa2 is present in all analyzed CA-MRSA strains, including the two previously sequenced ST59 CA-MRSA strains.

**Table 3:** Mobile genetic elements of main CA-MRSA lineages.

Genomic and pathogenicity islands	SA268 (ST59)	SA957 (ST59)	M013 (ST59)	FPR3757 (ST8)	MW2 (ST1)	11819-97 (ST80)
ΦSa2 (containing PVL)	+	+	+	+	+	+
νSa3	−	−	−	+	+	−
νSa4	−	−	−	+	+	−
νSaα	+	+	+	+	+	+
νSaβ	+	+	+	+	+	+
ΦSa3 (containing *sak*)	−	−	−	+	+	+
ACME	−	−	−	+	−	−
SCC*mec*	V	V(T)	V(T)	IV	IV	IV

As for pathogenicity islands, both strains FPR3757 and MW2 contain the νSa3 and νSa4 islands, which are absent from strains SA268, SA957, M013, and 11819-97. νSa3 contains two enterotoxin genes with unknown function in virulence, and νSa4 does not comprise known virulence factors.

Finally, USA300 strains (such as strain FPR3757) are the only *S. aureus* strains containing the arginine catabolic mobile element (ACME). It was recently reported that the virulence gene *speG* within ACME assists *S. aureus* to circumvent polyamine hypersensitivity and increase survival [22], [23]. The SA268 isolate does not contain ACME, in accordance with absence of this element from other sequenced ST59 strains [11], [12].

### Virulence Gene Analysis

We performed a genome-wide analysis to determine differences in virulence factor composition among CA-MRSA strains (Table 4). The overall composition of virulence genes among ST59 strains was identical: Strains SA268, SA957 and M013 all lack the *spl(s), sak, lytN, lukD/E, eta*, and *etb* genes, which encodes serine proteases, staphylokinase, cell wall hydrolase, leukotoxins, and exfoliative toxins, respectively. In contrast, these genes are present in strains FPR3757, MW2 and 11819-97.

**Table 4:** Differences in the presence of known virulence genes between ST59 and other CA-MRSA strains.

Annotation		SA26(ST59)	SA95(ST59)	M013(ST59)	FPR3757(ST8)	MW(ST1)	11819-97(ST80)
Serine proteases	*spl(s)*	−	−	−	+	+	+
Staphylokinase	*sak*	−	−	−	+	+	+
Cell wall hydrolase	*lytN*	−	−	−	+	+	+
Leukotoxin LukDE	*lukD,E*	−	−	−	+	+	+
Lantibiotic biosynthesis genes	*bsaA-G*	−	−	−	+	+	+
Exfoliative toxins	*eta,etb*	−	−	−	+	+	+
Collagen adhesion precursor	*cna*	−	−	−	−	+	−
*arcA* region of ACME	*ACME-arcA*	−	−	−	+	−	−

The genes coding for PVL, *lukS* and l*ukF*, are present in all CA-MRSA strains analyzed in this study. Among several putative determinants of CA-MRSA virulence, PVL has received most attention. It forms pores in the cell and mitochondrial membrane of neutrophils and macrophages and thus provokes cell lysis and apoptosis with subsequent liberation of inflammatory mediators [24], [25], [26], [27], [28]. While PVL facilitated experimental CA-MRSA lung and bone infection [29], [30], PVL did not have a significant impact on CA-MRSA virulence in several skin infection models [7], [31]. In addition, cases of PVL-negative CA-MRSA infections have been reported with increasing frequency [7]. Thus, the role of PVL in CA-MRSA infection remains controversial. Changes in the expression of key genome-encoded toxins such as α-toxin and phenol-soluble modulins (PSMs), were also proposed to have played a role in the evolution of virulence in CA-MRSA strains [32], [33], [34]. PSMs and α-toxin were shown to contribute significantly to several CA-MRSA disease manifestations, including SSTI, in animal infection models [35], [36]. As they are genome-encoded, α-toxin and PSM genes are present in all strains analyzed in our study.

### SCC*mec* Typing

**Figure 3:**
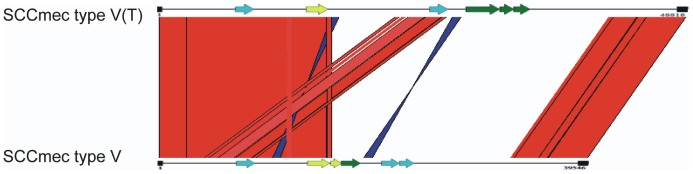
Structure and comparative analysis of SCC*mec* elements in the ST59 CA-MRSA strains. The SCC*mec* structure [SCC*mec* V(T)] of M013 and SA957 is shown in the upper part, and that of SA268 (SCC*mec* V) is shown in the lower part. Blue arrows, *ccr* operon; yellow arrows, *mec* operon; green arrows, *hsd* operon. Red and blue blocks represent the nucleotide alignment between the two SCC*mec* types.

CA-MRSA strains contain the low-fitness cost SCC*mec* elements of types IV or V [2], [6], [10]. The CA-MRSA strains FPR3757, MW2 and 11819-97 all carry SCC*mec* IV. In contrast, SA268 carries SCC*mec* V; and the other sequenced ST59 strains M013 and SA957 carry SCC*mec* V(T). A comparison of SCC*mec* elements of the three ST59 CA-MRSA strains is shown in Figure 3. The main difference between SCC*mec* V and SCC*mec* V(T) is that the latter possesses two distinct *ccrC* genes (*ccrC*1 allele 2 and allele 8) [10], [37].

### Comparative Genomics of ST59 CA-MRSA Strains

Next, we compared the genome of strain SA268 with those of strains M013 and SA957, which are ST59 CA-MRSA isolates from Taiwan. M013 was isolated from a wound infection [11] and SA957 from the blood of a patient suffering from a cutaneous abscess and bacteremia [12]. The three genomes were found to be highly similar to each other in terms of gene content. Nevertheless, SA268 contains specific segments within its SCC*mec* element, while SA957 and M013 have several mobile genetic elements and lipoproteins that are absent from strain SA268. Detailed information of gene difference is given in Table S1.

**Figure 4:**
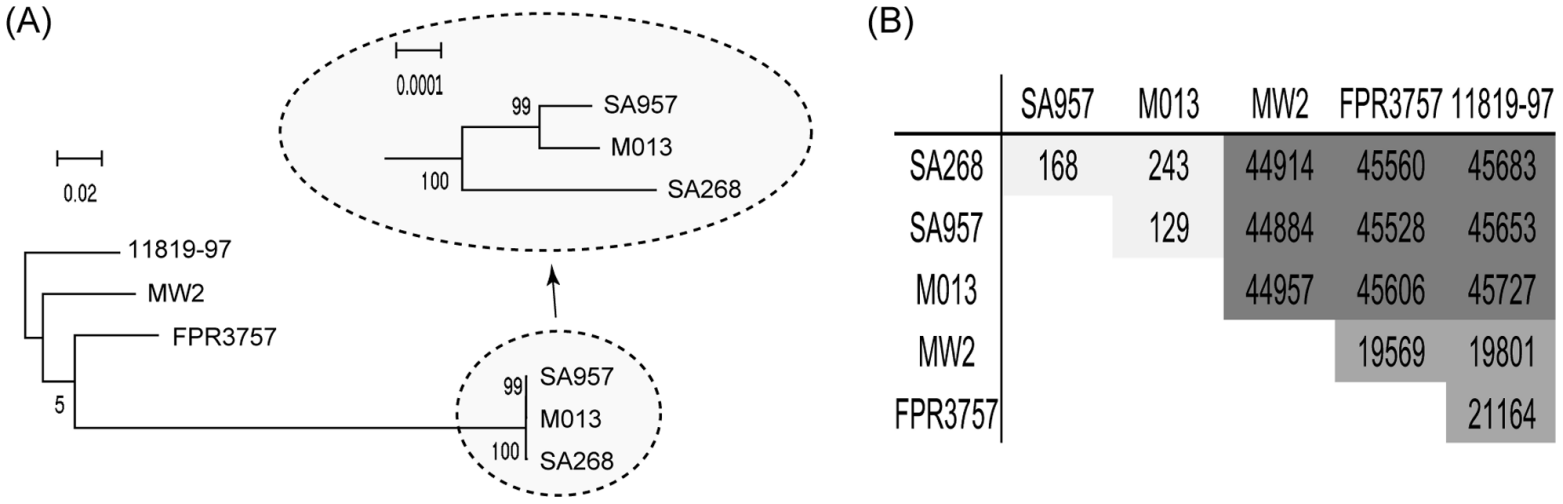
Evolutionary relationship of ST59 CA-MRSA genomes. (A) A Maximum-Likelihood tree was constructed based on conserved sequences in the genomes of ST59 strains SA268, M013 and SA957. The genomes of strains MW2, FPR3757 and 11819-97 were used as outgroups. The numbers near the nodes represent the SH-like support value. The scale bar is shown representing the genetic distance. (B) Number of pair-wise single nucleotide substitutions across the conserved genome.

To further infer the phylogenetic relationship between the three ST59 isolates, we constructed a Maximum-Likelihood (ML) tree based on conserved genome. The representative CA-MRSA strains, i.e. MW2, FPR3757 and 11819-97, were also included as controls. The regions which are conserved in these genomes total 2509374 bp, ca. 85% of the whole genome. The ML tree shows that SA268 is distinguished from the two Taiwanese isolates but the genetic distance is small (Figure 4). Within these conserved regions, the three ST59 isolates differs less than 250 Single Nucleotide Polymorphisms (SNPs), but at least 19000 SNPs are present between ST59 and non-ST59 CA-MRSA strains.

### Plasmids and Resistance Genes

SA268 contains one plasmid, named pSA268, which is almost identical to pPM1 from *S. aureus* PM1, a multidrug-resistant ST59 CA-MRSA strain from Taiwan [38]. A tetracycline resistance determinant, *tet(K)*, encoding a tetracycline efflux pump [39], [40], is located on pSA268. The *tet(K)*-carrying *S. aureus* strains have exclusively been described as tetracycline-resistant and minocycline-susceptible [39], [40]. However, strain SA268 was susceptible to tetracycline, which might be due to low-level expression of the *tet(K)* gene.

### Concluding Remarks

We here report a case of CA-MRSA necrotizing pneumonia and sepsis, severe manifestation of CA-MRSA disease that is still rare in China. We present the first sequenced genome of a PVL-positive ST59 CA-MRSA strain from the People’s Republic of China. The SA268 genome is almost identical to that of the major CA-MRSA lineage in Taiwan, which has the same sequence type, ST59. Small but noticeable differences are found in the SCC*me*c element and in gene composition. Notably, some lipoprotein genes found in the Taiwanese ST59 strains are absent from the Chinese ST59 strain. The CA-MRSA isolate from mainland China thus appears to be closely related, but still distinguishable from Taiwanese CA-MRSA strains. According to gene composition analysis, the assumed basis of virulence in the mainland Chinese CA-MRSA strain is to be explained in a similar fashion as reported for the Taiwanese strains, that is, by a combination of acquisition of the CA-MRSA-characteristic genes encoding PVL and SCC*mec* V, and a possibly increased expression of core genome-encoded virulence factors.

## Supporting Information

Figure S1 **PFGE patterns of the blood and sputum isolates.** Marker, *Salmonella enterica* serotype Braenderup strain H9812 DNA digested by XbaI, used as a molecular marker (kb); “Blood” and “Sputum” represent the blood isolate and the sputum isolate, respectively.
(TIF)

Table S1 **Comparison of gene content between the three ST59 strains.**
(XLS)
