# Supplementary figures and images for: Whole Genome Analysis of a Community-Associated Methicillin-Resistant Staphylococcus aureus ST59 Isolate from a Case of Human Sepsis and Severe Pneumonia in China
Source: PLoS One. 2014 Feb 20;9(2):e89235. doi: 10.1371/journal.pone.0089235 (PMC3930696; doi:10.1371/journal.pone.0089235)

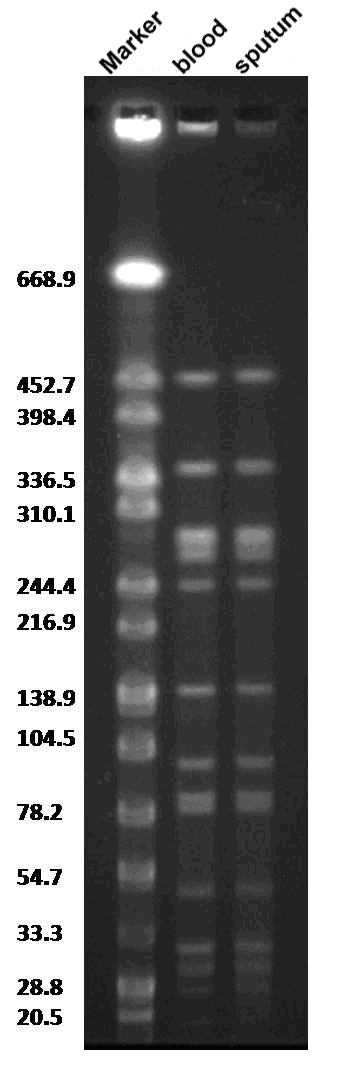

Supplement: Figure S1 — PFGE patterns of the blood and sputum isolates. Marker, Salmonella enterica serotype Braenderup strain H9812 DNA digested by XbaI, used as a molecular marker (kb); “Blood” and “Sputum” represent the blood isolate and the sputum isolate, respectively. (TIF) [file pone.0089235.s001.tif]
